# Fruit and vegetable intake in minority ethnic groups in the UK: analysis from ‘Understanding Society’ and UK Biobank

**DOI:** 10.1017/S136898002510102X

**Published:** 2025-08-27

**Authors:** Pooja Shah, Mayada Demashkieh, Basma Ellahi, Hibbah Osei-Kwasi, Sophia D. Amenyah, Reena Vijayakumaran, Jane Murphy, Rebecca Hardy

**Affiliations:** 1 Faculty of Health and Social Sciences, Bournemouth University, Bournemouth, UK; 2 School of Sports, Exercise and Health Sciences, Loughborough University, Loughborough, UK; 3 Faculty of Health, Medicine and Society, University of Chester, Chester, UK; 4 Faculty of Health and Life Sciences, Northumbria University, Newcastle upon Tyne, UK

**Keywords:** Dietary intake, Lifestyle and ageing, Ethnicity, United Kingdom

## Abstract

**Objective:**

To examine differences in fruit and vegetable intake and food insecurity between Black African and Caribbean and South Asian (Indian, Pakistani and Bangladeshi) ethnic minority groups with a White British/Irish reference population in the UK. This study was part of the TANGERINE project (nuTritional heAlth aNd aGeing in oldER ethnIc miNoritiEs).

**Design:**

Longitudinal analysis using multilevel logistic regression from Understanding Society and a cross-sectional comparison with UK Biobank.

**Setting:**

Understanding Society waves 2 (2010–2012), 5 (2013–2015), 7 (2015–2017), 9 (2017–2019) 11 (2019–2021) and 13 (2021–2023). UK Biobank baseline data (2006–2010).

**Participants:**

Understanding Society: adults aged 16 years and above (approximately 44 000 households). UK Biobank: participants aged 37–73 years (*n* = 502 412).

**Results:**

At wave 2, African, Caribbean, Pakistani and Bangladeshi participants in Understanding Society had lower odds of daily vegetable intake than White British/Irish participants, with Pakistanis showing the lowest intake. These disparities persisted after adjusting for socio-economic position (SEP) at individual and area levels, particularly for Caribbean and Pakistani groups. Indians consistently had higher odds of vegetable intake. Ethnic differences in fruit intake were smaller and largely attenuated by SEP adjustment. Food insecurity was more prevalent in all ethnic minority groups (except Indians) and associated with lower vegetable and fruit intake, though SEP explains more of the ethnic difference.

**Conclusions:**

Ethnic differences in fruit and vegetable intake are at least partially explained by SEP, with persistent vegetable consumption disparities after adjustment. Culturally tailored interventions addressing affordability, accessibility and SEP disparities are needed to improve dietary behaviours among minority ethnic groups.

The lifespan of the global population has been steadily increasing over decades, and the proportion of the global population of adults aged 65 years and over is projected to rise from 10 % in 2022 to 16 % by 2050^(1)^. In the United Kingdom (UK), the proportion of those in older age groups is growing rapidly, and it is predicted that a quarter of the population will be aged 65 years and over by 2050^(2)^. Compared with the increase in life years, there has been relatively little improvement in reducing the overall burden of age-related morbidity with more people with chronic conditions living longer^(3,4)^. The UK population is also becoming more ethnically diverse, with the largest increase since the last census in 2021 in Asians and Black African and Caribbean ethnic minority groups^(5)^. In the UK, both lifespan and years spent in good health are estimated to be lower in those from ethnic minority groups than White British/Irish people^(6)^.

Those from ethnic minority communities in the UK experience disproportionate health inequalities and may live in areas of higher deprivation and disadvantaged socio-economic position (SEP) compared with the White British/Irish population, predisposing them to poorer health and quality of life in older age^(6)^. Research shows there is high prevalence of early-onset obesity and other long-term conditions including type 2 diabetes, cardiovascular disease (CVD), and more recently COVID-19 in individuals from ethnic minority groups, with severe negative impacts on quality of life and health in older age^(6,7)^. For example, the Health Survey for England: ethnicity and health survey study, which was based on a sample of almost 74 000 adults and combined survey data from 2011 to 2019 reported that after accounting for age, hypertension was highest among Black Caribbean, Black African and Pakistani adults, and women from Black Caribbean (74 %), Pakistani (74 %) and Black African (73 %) backgrounds were most likely to be overweight or obese^(8)^. In a systematic review of the non-communicable disease burden among groups in high-income countries including the UK, prevalence of diabetes was shown to be consistently higher in all ethnic minority groups compared with the host populations^(9)^.

While recognising health disparities, factors that have been attributed to poor dietary health of adults from ethnic minority groups include financial constraints, language barriers, age, availability of traditional foods and years spent in the host country^(10)^. Qualitative research has shown that personal, social and cultural environmental factors influence eating behaviours and physical function in older adults, differing by age, ethnicity and gender^(11)^. Furthermore, it is often assumed that those from ethnic minority groups live in multigenerational households that offer continuous support for older members. However, growing evidence suggests that this is not the case, and that they do not have extensive social networks and are at increased risk of loneliness^(12)^. These factors have been amplified by the impacts of COVID-19 and the post-pandemic cost-of-living increases, increasing older people’s vulnerability to food insecurity and risk of poor nutrition through impact at household level. Age UK estimate that 2 million older adult households do not have sufficient funding to cover essential spending, with 26 % of those surveyed spending less on food shopping than previously^(13)^. Those from ethnic minority backgrounds are more likely to experience fuel poverty, and in particular, those from Black African communities are affected across multiple axes of vulnerability^(14,15)^, which may hinder people’s ability to prepare and cook hot food. A recently published Food Standards Agency report showed that food insecurity was more prevalent among South Asian, Black African and Black Caribbean individuals compared with White British/Irish groups of the population, including among older people^(16)^.

Promoting healthy ageing and increasing the amount of time spent in good health is an important priority, and recent studies have highlighted the role of healthy dietary patterns and food choices across the life course not just in mitigating adverse health conditions, but as a major modifiable factor to modulate the ageing process^(17)^. A nutritious diet, and consumption of fruit and vegetables in particular, is essential for physical and mental well-being^(18,19)^, for healthy ageing^(4,20)^ and for reducing the risk of physical long-term health conditions such as type 2 diabetes, CVD and obesity^(21,22)^. Due to its high nutritional benefits to health, the World Health Organization (WHO) recommend the consumption of at least 400 grams per day of fruit and vegetables^(23)^.

In 2003, the UK initiated a national ‘5-a-day’ campaign to encourage the public to eat at least five portions of fruit and vegetables a day, equating to roughly 400 g. However, despite a successful campaign, by 2022, fewer than a third of adults aged 16 years^(24)^ and over were meeting their goals, with the White British/Irish people more likely to do so than other ethnic groups, and women more likely than men^(25)^. Whilst some national surveys have reported the fruit and vegetable consumption patterns among different ethnic groups^(24–26)^, these vary in size, broad classification of ethnic groups, representation and size of ethnic minority group. Using robust national longitudinal datasets such as Understanding Society and UK Biobank to conduct secondary analyses provides an opportunity to address this research gap. Therefore, drawing on these datasets with a unique focus on heterogeneity of ethnic minority groups and comparison between ethnic groups, this study aims to compare fruit and vegetable intake between Black African and Caribbean and South Asian ethnic minority groups with the White British/Irish population in the UK and investigate the extent to which any differences are explained by markers of individual and area SEP. We also investigate the role of food insecurity on ethnic differences in fruit and vegetable intake.

## Methods

### Studies

Understanding Society^(27)^ is a nationally representative household panel survey started in 2009. It is based on clustered stratified probability sample of UK households, described in detail previously^(28)^, and initially included approximately 40 000 households. It includes members of the British Household Panel Study, which ran from 1991 to 2009 by the Institute for Social and Economic Research at the University of Essex and annually tracked changes in households and individuals from approximately 10 000 households over time across Great Britain. The general sample of Understanding Society included an ethnic minority boost sample of over 4000 households, and an immigrant and ethnic minority boost (IEMB) sample of approximately 2900 households was included from 2014/16 (wave 6). Data on participants are collected annually, and the most recent data were collected in 2021/23 (wave 13). Data from the main survey including participants 16 years and older was used in these analyses (UK Data Service Project Number 251326).

UK Biobank is a prospective epidemiological study, which initially included 502 412 participants aged 37–73 years. Participants were recruited from twenty-two assessment centres across England, Wales and Scotland, where baseline assessments took place between 2006 and 2010^(29)^. Information from the baseline questionnaires were used in the current study (UK Biobank Project Number 124326) as this provided the largest sample size including adequate numbers by ethnic group.

### Outcomes

In Understanding Society, fruit and vegetable intake was collected at waves 2 (2010–2012), 5 (2013–2015), 7 (2015–2017), 9 (2017–2019) 11 (2019–2021) and 13 (2021–2023) using a food frequency questionnaire (FFQ) asking how many times per week participants consumed each of these. For purposes of analysis, consumption every day was compared with the other three categories combined (never, 1–3 or 4–6 d per week), since recommendations are for fruit and vegetables to be eaten every day.

The United Nations Food Insecurity Experience Scale was administered at wave 13 and asked about food insecurity in the last 12 months. The series of eight questions were scored 1 if the participant answered yes and 0 for no. A sum was created from 0–8 and those with high food insecurity (4–8) were distinguished from some food insecurity (1–3) and none (0).

In the UK Biobank, data on fruit and vegetable intake were collected through a Touchscreen questionnaire administered during recruitment from 2006 to 2010. This questionnaire included questions about the frequency of consumption over the past year for cooked vegetables, salad/raw vegetables and fresh fruit. Participants reported their consumption by entering the number of heaped tablespoons (for vegetables) or pieces (for fruit) consumed per day. For analytical purposes, we standardised these measures by assuming that three heaped tablespoons of vegetables equated to one vegetable portion and one piece of fresh fruit equated to one fruit portion^(30)^. Participants were then categorised based on whether they consumed none or at least one portion of fruit daily. A similar categorisation was applied to vegetable intake.

### Ethnicity

Ethnicity in Understanding Society was self-identified using a modified version of the Office for National Statistics 2011 Census ethnic group question^(31)^. The ethnic groups for this analysis were White (British or Irish), Caribbean, African, Indian, Pakistani and Bangladeshi. While the numbers within these groups are smaller, we felt it was important to keep the groups separate, rather than considering Black African and Caribbean and South Asian, due to the different types of foods consumed within these larger groups. Individuals identifying as any other ethnicity were excluded from analyses.

Ethnicity data in the UK Biobank were self-reported during the initial Assessment Centre visit. For the purposes of our analysis, the ethnicity grouping was aligned with the categories used in the Understanding Society study^(32)^.

### Covariates

Income and a measure of disadvantage to the area of residence were included as markers of socioeconomic position.

In Understanding Society, estimated individual total net income, accounting for taxes on earnings and national insurance contributions, is provided as a derived variable at each wave. The variable is constructed as the sum of the six income components: labour, miscellaneous, private benefit, investment, pension and social benefit^(33)^ and was logged for analysis due to skewness. The level of disadvantage of the area in which the household was located was measured according to the index of multiple deprivation (IMD) categorised into equal fifths. For analyses, the three least disadvantaged groups were combined due to lack of numbers in these areas for ethnic minority groups.

In the UK Biobank, participants were asked about their household’s average total income before tax through the touchscreen question, ‘What is the average total income before tax received by your household?’ using a five-point scale. The Townsend deprivation index^(34)^, based on the national census output areas, was assigned according to postcode of residence.

### Statistical analysis

Primary analysis was carried out in Understanding Society given its national representativeness and the availability of fruit and vegetable intake at multiple timepoints. Fruit intake and vegetable intake were considered separately.

Repeated measures multilevel logistic regression with daily vegetable (or fruit) intake at waves 2, 5, 7, 9, 11 and 13 were carried out to assess how ethnic differences varied over time. While there was drop out from the study over time, these models included all those with at least one outcome measure. At wave 2, from a total of 50 444 participants with ethnicity, sex and age collected, 49 561 had at least one measure of vegetable intake, and 49 561 of fruit. Excluding those with missing income and IMD resulted in the analytic sample sizes of 48 641 for vegetable intake and 48 643 for fruit intake.

Waves were nested within individuals, with a random intercept, within household and sampling unit. These models account for the correlation between repeated measures on the same individuals by clustering. The analytic sample consisted of these providing fruit and vegetable information from wave 2 and, therefore, did not include the IEMB. Initial models included wave starting from wave 2, wave squared (as change over time was non-linear), age at wave 2, sex, age by wave interaction, and ethnic group. Sex by wave interactions was not included as they did not substantially improve the fit of the models for either vegetables or fruit. The interactions of wave by ethnicity and wave squared by ethnicity were then added to test whether differences in intake between ethnic minority groups compared with White British/Irish changed over time. The extent to which any differences in intake by ethnicity could be accounted for by SEP was assessed by additionally adjusting for own net weekly income and IMD of place of residence. Where differences across time were observed, estimated OR comparing each minority group with White British/Irish at each wave were obtained.

Cross-sectional complete case analyses of Understanding Society at wave 2 (2010–2012) and UK Biobank (2006–2010) were carried out as these data collections took place in a similar period. Logistic regression was used to assess the differences in vegetable or fruit intake by ethnic group, adjusted for age and sex. Adjustment for markers of SEP was then carried out. Analyses in Understanding Society were adjusted for clustered survey design, and relevant cross-sectional weights were used for representativeness^(35)^. These weights, along with guidance to selecting and applying them, are supplied by the Understanding Society Team^(35)^. The weights account for the complex survey design enabling the findings to be generalisable to the UK population at the time of wave 2 data collection.

Multinomial logistic regression, accounting for stratified sampling and weighted for population representativeness, was used to assess ethnic group differences in food insecurity (in three categories) at wave 13, adjusted initially for age and sex. The models were then adjusted for own net income and IMD. The analytical sample included all participants responding to the relevant questions at wave 13 and thus did include the IEMB as well as the ethnic minority boost . In addition, logistic regression models were run with daily vegetable or fruit intake as the outcome. A series of models were fitted including (a) age, sex, food insecurity; (b) age, sex, ethnic group, food security; (c) age, sex, ethnic group, SEP and (d) age, sex, ethnic group, food insecurity, SEP. Food insecurity by ethnic group interactions assessed whether there was ethnic variation in associations in model b.

### Supplementary analysis

As the main longitudinal analysis in Understanding Society did not include the IEMB, we carried out supplementary analyses, repeating the longitudinal modelling from wave 7 when the IEMB is first included using the relevant sampling and household units from wave 7. Only linear change with wave was fitted given there are only 4 waves included in those models.

## Results

### Ethnic differences in vegetable and fruit intake in Understanding Society

In wave 2 of Understanding Society, of those with ethnicity data, 48 213 adults answered the vegetable intake question and 48 214 the fruit intake question, regardless of whether they reported consuming any or not (Table 1). The weighted estimated percentages eating both vegetables and fruit every day were lower in African, Caribbean, Pakistani and Bangladeshi groups compared with the White British/Irish. Pakistanis had the lowest percentage eating vegetables every day (23 %) and Bangladeshis the lowest for fruit (31 %) (Table 1). Indians had very similar patterns of intake to the White British/Irish group. At wave 13, a decrease in the proportions eating vegetables and fruit every day was observed in all ethnic groups (Table 1). For vegetables, the decrease was substantial in some groups (e.g. 51 % to 36 % in White British/Irish and 38 to 26 % in Africans), and there was also an increase in the percentage reporting never eating them (e.g. 2 % to 10 % in Pakistani).

**Table 1.** Estimated percentages of vegetable and fruit intake by ethnic group at wave 2 and wave 13 in Understanding Society (total available sample size at each wave)

		Wave 2					Wave 13			
	Observed *n*	Never (%)	1–3 d/week (%)	4–6 d/week (%)	Every day (%)	Observed *n*	Never (%)	1–3 d/week (%)	4–6 d/week (%)	Every day (%)
Vegetables										
White British/Irish	42 784	2	19	27	51	23 197	8	34	22	36
Caribbean	1040	4	37	23	36	421	7	44	20	29
African	864	3	33	26	38	454	7	44	23	26
Indian	1510	1	24	25	49	1081	8	39	24	30
Pakistani	1184	2	48	26	23	951	10	49	21	20
Bangladeshi	831	2	39	21	39	496	7	48	22	22
Fruit										
White British/Irish	42 786	8	28	19	46	23 205	3	26	31	40
Caribbean	1041	5	42	18	36	422	6	44	22	28
African	863	5	40	19	36	456	6	40	25	30
Indian	1510	4	30	20	47	1083	5	34	28	34
Pakistani	1184	6	36	18	40	962	11	62	14	13
Bangladeshi	830	4	47	18	31	497	9	42	26	23

**Figure 1.**
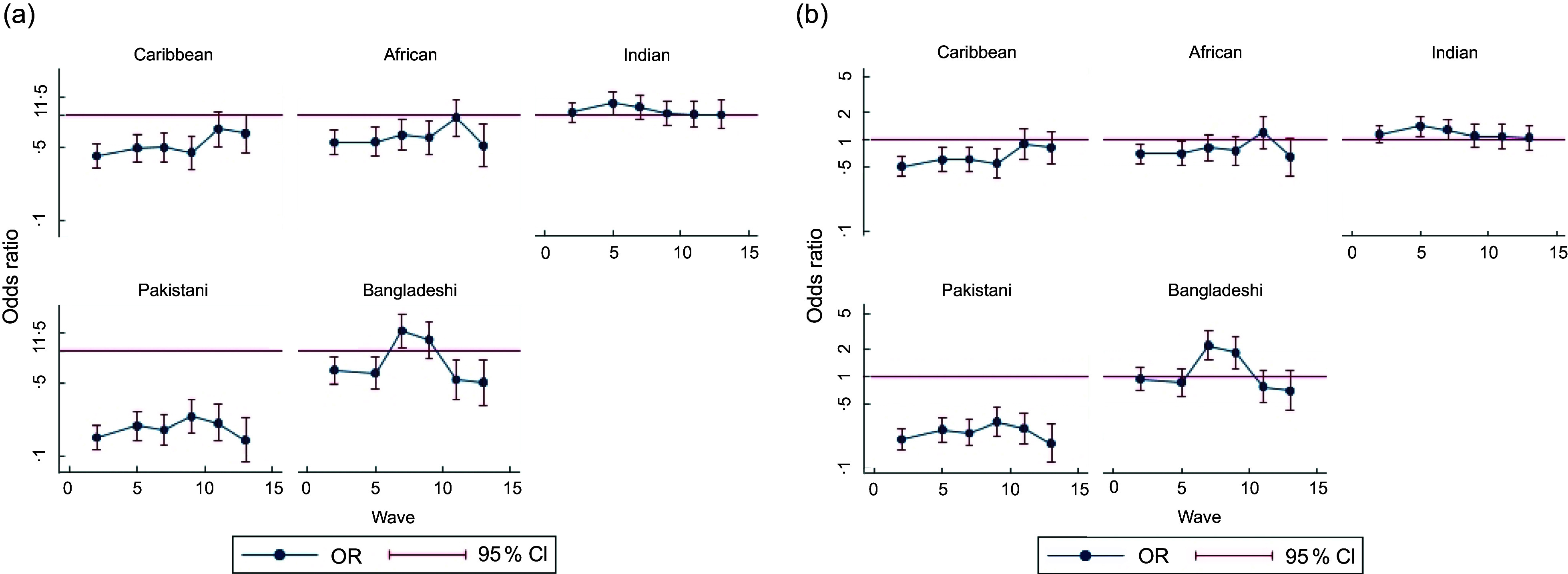
Estimated OR for each wave for each ethnic minority group compared with white British/Irish group from longitudinal models (including wave, wave by age and ethnic group) for vegetable intake from waves 2, 5, 7, 9, 11 and 13 of Understanding Society. (a) Adjusted for age, sex; (b) adjusted for age, sex, net income and IMD. IMD, index of multiple deprivation.

The analytic sample size of those with at least one measure of the outcome and complete covariate information was 48 641 for vegetable intake and 48 643 for fruit intake. In sex- and age-adjusted longitudinal models, all ethnic minority groups except Indians had lower odds of daily vegetable intake than White British/Irish at wave 2, with the difference being greatest for the Pakistani group (OR (95 % CI) = 0·14 (0·11, 0·19)) (Table 2). The odds of daily vegetable intake followed a non-linear pattern from waves 2 to 13, with Indian, Pakistani and Bangladeshi groups showing different trends compared with the White British/Irish group (Table 2, see online supplementary material, Supplemental Figure 1(a)). While White British/Irish, Caribbean and African Groups showed a similar decline starting from wave 5, the decline began slightly later for Indian and Bangladeshi groups and was more pronounced among the Pakistani group. The OR for African and Indian groups compared with the White British/Irish remained reasonably stable over time (Figure 1(a)). For example, for the African group, the OR (95 % CI) was 0·55 (0·42, 0·70) at wave 2 and 0·51 (0·31, 0·82) at wave 13. The difference in intake between Caribbeans and White British/Irish reduced at the more recent waves, while that between Pakistani and White British/Irish increased. The odds of daily vegetable intake were higher for the Bangladeshis than the White British/Irish at waves 5 and 7, but lower again at subsequent waves (Figure 1(a)).

**Table 2.** Results from longitudinal models for ethnic differences in daily vegetable and fruit intake at waves 2, 5, 7, 9, 11 and 13

	Vegetables (48 641 individuals and 170 979 observations)				Fruit (48 643 individuals and 170 990 observations)			
	Adjusted for age, sex, age × time		+net income and IMD		Adjusted for age, sex, age × wave		+net income and IMD	
	OR	95 % CI	OR	95 % CI	OR	95 % CI	OR	95 % CI
Wave 2 (baseline)								
White British/Irish	1		1		1		1	
Caribbean	0·41	0·32, 0·53	0·52	0·41, 0·67	0·52	0·40, 0·67	0·64	0·50, 0·82
African	0·52	0·40, 0·67	0·67	0·53, 0·86	0·86	0·67, 1·09	1·07	0·84, 1·37
Indian	1·06	0·86, 1·31	1·17	0·95, 1·44	1·50	1·22, 1·84	1·64	1·34, 2·01
Pakistani	0·14	0·11, 0·19	0·20	0·15, 0·26	0·90	0·71, 1·14	1·23	0·98, 1·56
Bangladeshi	0·58	0·43, 0·78	0·85	0·63, 1·14	0·51	0·38, 0·68	0·72	0·53, 0·96
Linear change over time (per year)								
White British/Irish	1·00	0·99, 1·02	1·00	0·99, 1·01	1·01	0·99, 1·03	1·03	1·02, 1·04
Caribbean	1·03	0·94, 1·13	1·03	0·93, 1·12	1·21*	1·10, 1·33	1·20*	1·09, 1·31
African	1·08	0·98, 1·19	1·07	0·97, 1·18	1·14^	1·04, 1·26	1·13^	1·03, 1·24
Indian	1·06	0·99, 1·14	1·06^	0·99, 1·14	1·06	0·99, 1·14	1·06	0·99, 1·13
Pakistani	1·16*	1·05, 1·27	1·15*	1·05, 1·27	1·11	1·03, 1·21	1·11	1·02, 1·20
Bangladeshi	1·30*	1·17, 1·44	1·29*	1·17, 1·43	1·21*	1·09, 1·35	1·20*	1·08, 1·33
Quadratic change over time (pear year^2^)								
White British/Irish	0·992	0·991, 0·993	0·992	0·991, 0·994	0·991	0·988, 0·991	0·992	0·991, 0·993
Caribbean	0·994	0·985, 1·003	0·994	0·986, 1·003	0·979*	0·970, 0·988	0·980*	0·970, 0·989
African	0·987	0·978, 0·996	0·988	0·978, 0·997	0·985	0·976, 0·995	0·986	0·976, 0·995
Indian	0·986^	0·979, 0·992	0·985*	0·979, 0·992	0·987	0·980, 0·993	0·987	0·980, 0·993
Pakistani	0·980*	0·970, 0·989	0·979*	0·970, 0·989	0·985	0·977, 0·993	0·985	0·977, 0·993
Bangladeshi	0·966*	0·957, 0·976	0·967*	0·957, 0·977	0·980*	0·969, 0·990	0·980*	0·970, 0·990

IMD, index of multiple deprivation. *p < 0.05 compared with White British/Irish; ^*p* < 0.1 compared with White British/Irish.

After adjusting for SEP, differences at wave 2 for Caribbean, African, Pakistani and Bangladeshis (Table 2) were attenuated, although differences remained for the Caribbean (OR (95 % CI) = 0·52 (0·41, 0·67)) and Pakistani (OR (95 % CI) = 0·20 (0·15, 0·26)) groups. Indians had higher odds of daily vegetable intake compared with White British/Irish after SEP adjustment at wave 2 (OR (95 % CI) = 1·17 (0·95, 1·44)). By wave 13, there was no evidence of a difference in daily vegetable intake (Figure 1(b)).

**Figure 2.**
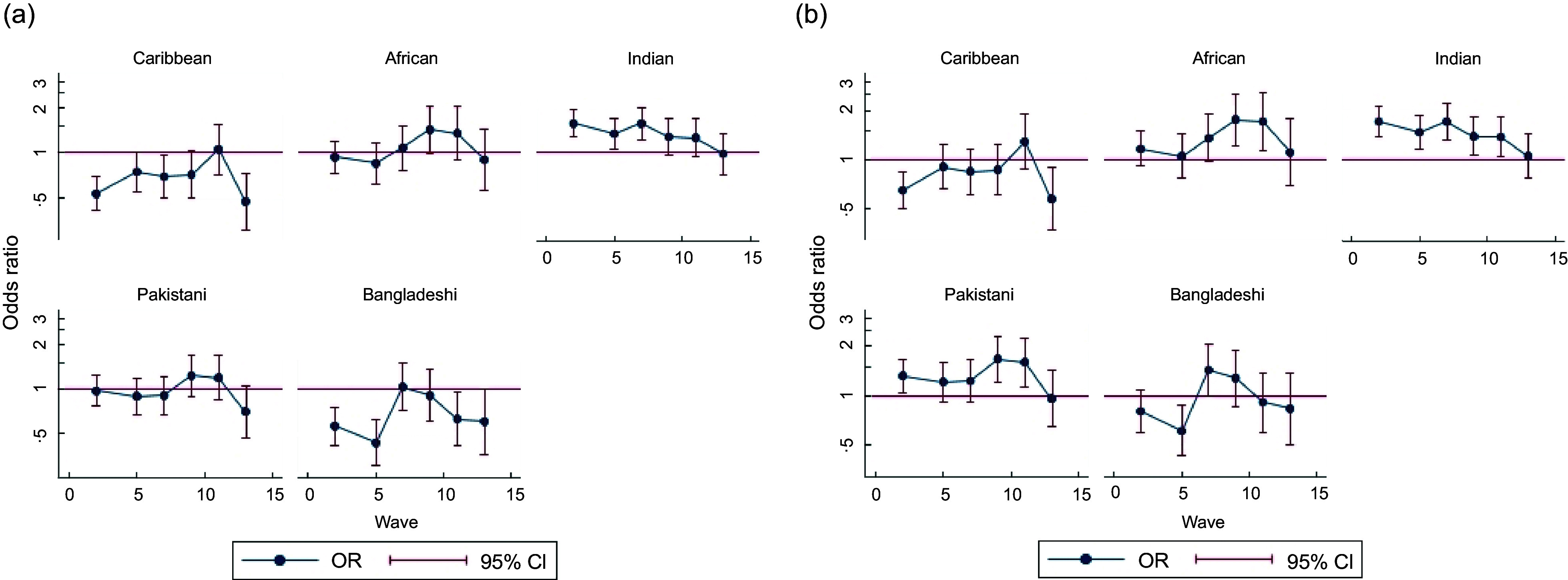
Estimated OR for each wave for each ethnic minority group compared with white British/Irish group from longitudinal models (including wave, wave by age and ethnic group) for fruit intake from waves 2, 5, 7, 9, 11 and 13 of Understanding Society. (a) Adjusted for age, sex; (b) adjusted for age, sex, net income and IMD. IMD, index of multiple deprivation.

In age- and sex-adjusted models, the ethnic minority differences in daily fruit intake were smaller than for vegetable intake at wave 2. Caribbean (OR (95 % CI) = 0·52 (0·40, 0·67)) and Bangladeshi (OR (95 % CI) = 0·51 (0·38, 0·68)) groups had lower odds of daily fruit intake compared with White British/Irish, while the odds for fruit intake among Indians (OR (95 % CI) = 1·50 (1·22, 1·84)) was higher. There was no evidence of a difference between Africans or Pakistanis and White British/Irish (Table 2). Change in odds of daily fruit intake demonstrated a non-linear pattern from waves 2 to 13, which varied across ethnic groups (Table 2, see online supplementary material, Supplemental Figure 2(a)). The estimated OR at each wave for Caribbean and Bangladeshi groups compared with White British/Irish decreased towards an OR of 1 and then increased again by wave 13 (Figure 2(a)). The higher odds of daily fruit intake at wave 2 in Indians compared with White British/Irish declined so that by wave 13 there was no difference (OR (95 % CI) = 0·98 (0·71, 1·35)), while the OR for African and Pakistani groups remained similar to the White British/Irish over time (Figure 2(a)).

After adjusting for SEP, African and Pakistani in addition to the Indian group generally had greater odds of daily intake of fruit than the White British/Irish, except at wave 13 when intake was similar (Figure 2(b)). Adjustment for SEP attenuated the OR for Caribbeans, and the Bangladeshi group had higher odds of daily intake at waves 7 and 9 but had similar intake at other waves (Figure 2(b)).

### Cross-sectional analyses of Understanding Society and UK Biobank

Consistent with the multilevel models, after adjustment for age and sex, the odds of consuming vegetables every day at wave 2 (*n* = 46 080) was lower in African, Caribbean, Pakistani and Bangladeshi groups compared with White British/Irish (Table 2). Adjustment for SEP explained some of the ethnic differences, but differences remained for Caribbean and Pakistani groups. Additional analysis adjusting separately for own net income and IMD suggests that IMD has the greater impact on estimates with addition of net income having only a minor impact once IMD is in the model. The ethnic differences in vegetable intake in UK Biobank (*n* = 395 567) were smaller than for Understanding Society, with Indians more likely to eat a portion of vegetables every day than the White British/Irish (OR (95 % CI) = 1·48 (1·35, 1·61)) and no difference observed for Bangladeshis (OR (95 % CI) = 0·84 (0·57, 1·22)) (Table 3). The lower odds for all groups except Indians were largely explained by SEP.

**Table 3.** Association between ethnic group and vegetable and fruit daily intake in the UK Biobank – adjusted for age and sex and additionally adjusted for household income and area deprivation. Sample size is number with information on all variables in SEP adjusted model

	Understanding Society Wave 2 (vegetables *n* = 46 080 fruit *n* 46 081)				UK Biobank (vegetables *n* = 395 567, fruit *n* = 399 975)			
	Adjusted for age and sex		+net income and IMD		Adjusted for age and sex		+household income and Townsend	
	OR	95 % CI	OR	95 % CI	OR	95 % CI	OR	95 % CI
Vegetables								
White British/Irish	1		1		1		1	
Caribbean	0·58	0·49, 0·69	0·70	0·60, 0·83	0·87	0·80, 0·94	1·06	0·98, 1·16
African	0·70	0·59, 0·83	0·87	0·73, 1·04	0·66	0·60, 0·72	0·88	0·80, 0·96
Indian	1·09	0·94, 1·27	1·15	0·99, 1·35	1·48	1·35, 1·61	1·59	1·46, 1·74
Pakistani	0·33	0·26, 0·41	0·43	0·34, 0·54	0·89	0·78, 1·02	1·13	0·98, 1·29
Bangladeshi	0·81	0·64, 1·02	1·06	0·84, 1·34	0·84	0·57, 1·22	1·17	0·80, 1·73
Fruit								
White British/Irish	1		1		1		1	
Caribbean	0·69	0·58, 0·81	0·80	0·67, 0·94	1·43	1·28, 1·59	1·35	1·20, 1·51
African	0·92	0·78, 1·09	1·09	0·92, 1·30	1·41	1·24, 1·61	1·91	1·66, 2·19
Indian	1·37	1·19, 1·58	1·44	1·24, 1·67	1·83	1·65, 2·04	1·80	1·60, 2·02
Pakistani	1·01	0·85, 1·20	1·29	1·08, 1·54	1·51	1·27, 1·79	1·91	1·58, 2·32
Bangladeshi	0·75	0·58, 0·97	0·94	0·72, 1·22	1·25	0·78, 2·02	2·06	1·20, 3·56

SEP, socio-economic position; IMD, index of multiple deprivation.

For daily fruit intake (*n* = 46 081), the odds were lower in only Caribbean and Bangladeshi groups compared with White British/Irish, with Indians having higher odds. SEP adjustment explained most of the observed differences. As for vegetable intake, IMD explains more of the ethnic differences than own net income. In contrast to Understanding Society, all ethnic minority groups in UK Biobank (*n* = 399 975) had higher odds of daily fruit consumption than the White British/Irish group before adjustment for SEP in UK Biobank (Table 3). Adjusting for SEP further increased the OR for all, except the Bangladeshi, groups to around 2.

### Ethnic differences in food insecurity and contribution to vegetable and fruit intake

All ethnic minority groups, except Indians, were more likely to report greater food insecurity than White British/Irish, with relative risk ratios (RRR) of around 2 or above for moderate food insecurity (scores 1–3) (Table 4). Africans have a particularly high RRR for high levels of food insecurity (score of 4+) (RRR (95 % CI) = 2·43 (1·48, 3·99)). The greater food insecurity was at least partially explained by SEP. RRR remained higher for African and Bangladeshi groups (moderate insecurity only).

**Table 4.** Association between ethnic group and food insecurity at wave 13 of Understanding Society – adjusted for age and sex and additionally adjusted for own and area socio-economic position. Estimates from logistic regression model with weighting

	Adjusted for age and sex				+net income and IMD			
	1–3		4+		1–3		4+	
	RRR	95 % CI	RRR	95 % CI	RRR	95 % CI	RRR	95 % CI
*n* 24 272								
White British/Irish	1		1					
Caribbean	1·97	1·08, 3·62	2·43	1·48, 3·99	1·47	0·80, 2·70	1·58	0·96, 2·61
African	2·94	1·69, 5·13	4·62	2·73, 7·82	2·16	1·24, 3·78	2·91	1·70, 5·00
Indian	1·33	0·89, 1·99	0·95	0·46, 1·98	1·22	0·81, 1·83	0·83	0·41, 1·69
Pakistani	1·93	1·28, 2·90	1·80	0·94, 3·47	1·30	0·86, 1·99	1·01	0·51, 1·97
Bangladeshi	4·35	2·15, 8·82	2·10	1·19, 3·69	2·76	1·39, 5·51	1·05	0·58, 1·92

IMD, index of multiple deprivation; RRR, relative risk ratios.

Greater food insecurity was associated with lower odds of daily vegetable (OR (95 % CI) = 0·31 (0·24, 0·40) for 4 + *v*. 0) and fruit (OR (95 % CI) = 0·29 (0·22, 0·38) for 4 + *v*. 0) intake, both before (see online supplementary material, Supplemental Table 4) and after inclusion of ethnic group in the model (Table 5 model b). Food insecurity explains less of the ethnic group differences in vegetable and fruit intake than SEP (Table 5 model c compared with model b). Addition of food insecurity after net income and IMD attenuated the estimates only slightly (Table 5 model d compared with model c). For vegetable intake, there was no evidence of an interaction between ethnic group and food insecurity. There was, however, suggestion that the association between food insecurity and fruit intake was weaker in Bangladeshi and Pakistani groups compared with White British/Irish.

**Table 5.** Logistic regression models (weighted) investigating the association between food insecurity and fruit and vegetable intake in wave 13 of understanding society

	Wave 13 (vegetables *n* = 24 161, fruit *n* = 24 171)					
	Age, sex, food insecurity (model b)		Age, sex, net income and IMD (model c)		Age, sex, net income, IMD and food insecurity (model d)	
	OR	95 % CI	OR	95 % CI	OR	95 % CI
Vegetables						
White British/Irish	1		1		1	
Caribbean	0·77	0·56, 1·06	0·91	0·65, 1·27	0·94	0·67, 1·33
African	0·82	0·53, 1·26	0·93	0·60, 1·43	1·03	0·66, 1·61
Indian	0·94	0·73, 1·22	1·00	0·76, 1·31	0·99	0·76, 1·30
Pakistani	0·27	0·19, 0·39	0·36	0·25, 0·53	0·37	0·25, 0·53
Bangladeshi	0·68	0·49, 0·94	0·92	0·66, 1·28	0·96	0·69, 1·35
Food insecurity score 0	1				1	
1–3	0·60	0·50, 0·72	–	–	0·67	0·56, 0·81
4+	0·31	0·24, 0·41	–	–	0·37	0·28, 0·49
Fruit						
White British/Irish	1		1		1	
Caribbean	0·76	0·54, 1·06	0·86	0·60, 1·22	0·89	0·63, 1·28
African	1·13	0·78, 1·64	1·22	0·83, 1·78	1·37	0·93, 2·01
Indian	0·98	0·78, 1·24	1·03	0·81, 1·31	1·03	0·81, 1·31
Pakistani	0·75	0·55, 1·04	0·96	0·70, 1·33	0·99	0·71, 1·38
Bangladeshi	0·81	0·53, 1·23	1·04	0·68, 1·58	1·09	0·70, 1·69
Food insecurity score 0	1				1	
1–3	0·63	0·52, 0·77	–	–	0·69	0·57, 0·84
4+	0·29	0·22, 0·38	–	–	0·34	0·25, 0·45

IMD, index of multiple deprivation.

### Supplementary analysis

Findings for the analysis starting from wave 7 including the IEMB sample are consistent with the main longitudinal analysis for vegetable and fruit intake (see online supplementary material, Supplemental Table 5).

## Discussion

This study revealed significant disparities in fruit and vegetable intake, with African, Caribbean, Bangladeshi and Pakistani groups consuming less than the White British/Irish population, while intake among the Indian groups was comparable. Between 2010 and 2012 (wave 2), the lowest daily vegetable intake was seen in Pakistanis and the lowest fruit intake in Bangladeshis. Over time, there was a general decline in fruit and vegetable intake across all groups by 2021–2023 (wave 13), with Pakistanis showing the largest decrease in intake. While ethnic differences in vegetable intake were attenuated after adjusting for SEP, substantial disparities remained for Caribbean and Pakistani groups. For fruit intake, differences were smaller but still notable, with Caribbean and Bangladeshi groups reporting lower intake compared with White British/Irish, while Indians had higher odds of daily fruit consumption. Adjustment for SEP largely explained these differences. Although greater food insecurity was also related to lower vegetable and fruit intake, it did not explain much for the ethnic differences in intake. Analysis of the UK Biobank revealed smaller disparities, with SEP explaining much of the difference. These findings underscore the complex role of ethnicity, SEP and food insecurity in dietary patterns.

Intake of fruits and vegetables has been shown to be associated with SEP^(36,37)^, and the decline across all ethnic groups over time suggests that socio-economic pressures as well as structural or environmental factors may be influencing dietary habits. The combined effect of Brexit and the COVID-19 pandemic, both of which occurred between the two waves of data collection, resulted in an abnormal inflation in the cost of fruit and vegetables^(38)^. Other possible explanations could include reduced access to affordable fresh produce, shifts in cultural food preferences influenced by local food environments or broader socio-economic pressures^(39)^. The greater reduction among Pakistani and White British/Irish groups may reflect unique challenges faced by these populations, such as neighbourhood food availability or changing family dynamics that influence meal planning and preparation^(39,40)^.

Vegetable and fruit intake show distinct patterns between ethnic groups, with vegetable intake disparities being more pronounced than fruit intake. SEP adjustments reduce the disparities in fruit intake to a greater extent than for vegetables, possibly because they directly address the affordability, accessibility and convenience barriers that disproportionately affect fruit consumption, suggesting that vegetable intake may be further influenced by cultural dietary habits and the higher cost or limited availability of diverse, fresh vegetables in some areas. For example, Pakistani meals are often meat based, with vegetables being presented as a side dish^(41)^, whereas many Indians are vegetarian, and many Indian dishes are centred around vegetables^(42,43)^. Additionally, fruits are often more convenient to consume without preparation, which may contribute to more consistent intake. Health messaging also tends to promote fruit as a snack^(44)^, making it easier for individuals to incorporate, while vegetables are often recommended as part of full meals, which could be less accessible for certain groups, especially in food-insecure or lower-income households.

Whereas previous research has focused on dietary differences of larger categories of ethnic minority groups such as South Asians or Afro-Caribbeans compared with White British adult populations^(45,46)^, there is substantial variation in diets within these groups, such as between the Pakistani, Bangladeshi and Indian groups. This study was conducted as part of a larger funded research project to improve nutritional health in older adults (TANGERINE: nuTritional heAlth aNd aGeing in oldER ethnIc miNoritiEs; https://www.bournemouth.ac.uk/research/projects/tangerine; ISRCTN 71774112) and highlights the heterogeneity within different ethnicities and explores how fruit and vegetable consumption patterns vary between different minority ethnic groups in the UK.

While this study provides valuable insights into dietary changes over time, some limitations should be acknowledged. Our longitudinal models assumed that household membership remained constant from the baseline wave included in longitudinal analysis and thus does not account for household moves. This assumption may have introduced misclassification, potentially affecting the accuracy of observed dietary patterns over time. This also meant we did not include the IEMB who joined at wave 7, but we did carry out supplementary analyses from wave 7 and findings were very similar to the main analysis. The use of multilevel modelling that allows the inclusion of all those with at least one measure of the outcome (under the missing at random assumption) means that the impact of missing data is likely to be small. However, it is possible that missing data are non-ignorable and therefore we cannot rule out the possibility of bias in the estimates of ethnic differences. UK Biobank is not representative of the UK population, and this may be an explanation for the smaller ethnic differences in vegetable and fruit intake compared with Understanding Society. This lack of representativeness could limit the generalisability of our findings to the broader UK population. The results from Understanding Society are more likely than those from UK Biobank to reflect generalisable findings, particularly given the use of weights. The datasets used in the study were not developed to suit cultural needs, and the tools may not appropriately capture individual differences that vary within ethnic groups. The outcome measure was based on whether individuals consumed at least one portion of vegetables or fruit a day, rather than assessing adherence to the recommended five-a-day guideline. This constraint reflects the structure of the survey questions in the datasets. As such, while the analysis provides insight into daily consumption patterns, it may underestimate the extent of inadequate fruit and vegetable intake across all groups. Previous research suggests that adherence to the five-a-day recommendation varies by ethnicity^(25)^. Therefore, the disparities observed in this study may underrepresent the true scale of ethnic differences in meeting national dietary guidelines. Self-reported dietary data are subject to reporting biases, including social desirability and recall biases, which may vary across different populations. Language barriers and varying levels for engagement could also have influenced the accuracy of responses, particularly among participants from diverse backgrounds. There could also be confusion in answering some survey questions such as vegetables being measured as spoonful amounts in the UK Biobank. The potential biases and measurement errors highlighted may have influenced the observed associations, and thus, conclusions drawn from this study should consider these factors.

Future research should focus on identifying specific cultural, social and environmental influences on diet that may be unique to different ethnic groups and how these change over time. Additionally, further studies could also investigate how specific dimensions of socio-economic disadvantage and food insecurity interact with food choices and create barriers to fruit and vegetable consumption among particular ethnic groups such as Pakistanis and Bangladeshis. There is a need to develop culturally tailored public health interventions and policies to improve nutritional health among some ethnic minority groups and to foster healthy dietary behaviours that support healthy ageing. However, engagement of individuals from these groups in research remains low, highlighting the importance of using diverse approaches such as coproduction to effectively reach and involve underserved communities.

## Supporting information

Shah et al. supplementary material Shah et al. supplementary material

## Supplementary material

For supplementary material accompanying this paper visit https://doi.org/10.1017/S136898002510102X
