# Supplementary material for: Fruit and vegetable intake in minority ethnic groups in the UK: analysis from ‘Understanding Society’ and UK Biobank
Source: Public Health Nutr. 2025 Aug 27;28(1):e159. doi: 10.1017/S136898002510102X (PMC12516610; doi:10.1017/S136898002510102X)
Supplement: Shah et al. supplementary material [file S136898002510102Xsup001.docx]

Supplementary Table 1: Percentages of individuals eating at least 1 portion of vegetables and fruits per day by ethnic group in the UK Biobank

|  | **Observed n** | **0 portions/day** | **≥ 1 portion/day** |
| --- | --- | --- | --- |
| **Vegetables** |  |  |  |
| White British/Irish | 448 310 | 17.7 | 82.3 |
| Caribbean | 4 277 | 10.5 | 79.5 |
| African | 3 098 | 29.1 | 70.9 |
| Indian | 5 593 | 13.7 | 86.3 |
| Pakistani | 1 654 | 25.2 | 74.8 |
| Bangladeshi | 209 | 23.4 | 76.6 |
| **Fruit** |  |  |  |
| White British/Irish | 454 536 | 9.47 | 90.53 |
| Caribbean | 4 426 | 9.40 | 90.60 |
| African | 3 285 | 9.41 | 90.59 |
| Indian | 5 861 | 6.93 | 93.07 |
| Pakistani | 1 795 | 9.86 | 90.14 |
| Bangladeshi | 223 | 10.76 | 89.24 |

Supplementary Table 2: Estimates of OR at each wave (these are presented in Figures 1a and 2a)

|  | **Wave** | | | | | |
| --- | --- | --- | --- | --- | --- | --- |
|  | **2** | **5** | **7** | **9** | **11** | **13** |
| **Vegetables** |  |  |  |  |  |  |
| White British/Irish | 1 | 1 | 1 | 1 | 1 | 1 |
| Caribbean | 0.41 (0.31, 0.53) | 0.48 (0.36, 0.66) | 0.49 (0.35, 0.68) | 0.44 (0.31, 0.63) | 0.73 (0.50, 1.08) | 0.66 (0.44, 1.01) |
| African | 0.55 (0.42, 0.70) | 0.56 (0.41, 0.76) | 0.61 (0.43, 0.87) | 0.61 (0.42, 0.88) | 0.94 (0.63, 1.40) | 0.51 (0.31, 0.82) |
| Indian | 1.05 (0.85, 1.31) | 1.29 (1.00, 1.65) | 1.19 (0.91, 1.54) | 1.03 (0.78, 1.36) | 1.01 (0.75, 1.36) | 1.00 (0.73, 1.37) |
| Pakistani | 0.15 (0.11, 0.20) | 0.19 (0.14, 0.26) | 0.18 (0.13, 0.25) | 0.24 (0.16, 0.34) | 0.20 (0.14, 0.30) | 0.14 (0.09, 0.27) |
| Bangladeshi | 0.65 (0.48, 0.88) | 0.61 (0.43, 0.87) | 1.53 (1.05, 2.24) | 1.27 (0.85, 1.88) | 0.53 (0.35, 0.80) | 0.50 (0.30, 0.82) |
| **Fruit** |  |  |  |  |  |  |
| White British/Irish | 1 | 1 | 1 | 1 | 1 | 1 |
| Caribbean | 0.53 (0.41, 0.68) | 0.74 (0.55, 1.01) | 0.69 (0.50, 0.96) | 0.71 (0.49, 1.02) | 1.05 (0.71, 1.56) | 0.47 (0.30, 0.73) |
| African | 0.93 (0.72, 1.19) | 0.84 (0.62, 1.15) | 1.07 (0.77, 1.50) | 1.42 (0.99, 2.05) | 1.35 (0.90, 2.02) | 0.89 (0.56, 1.42) |
| Indian | 1.57 (1.27, 1.93) | 1.34 (1.05, 1.70) | 1.56 (1.21, 2.02) | 1.28 (0.97, 1.68) | 1.26 (0.94, 1.68) | 0.98 (0.71, 1.35) |
| Pakistani | 0.97 (0.77, 1.24) | 0.90 (0.68, 1.19) | 0.91 (0.68, 1.22) | 1.23 (0.89, 1.70) | 1.20 (0.84, 1.70) | 0.71 (0.47, 1.05) |
| Bangladeshi | 0.57 (0.42, 0.76) | 0.44 (0.31, 0.62) | 1.03 (0.71, 1.50) | 0.91 (0.61, 1.35) | 0.63 (0.42, 0.96) | 0.61 (0.37, 1.01) |

Supplementary Table 3: Estimated percentages (accounting for weighting) for food insecurity questions by ethnic group in wave 13 in Understanding Society

|  | **Observed n** | **0** | **1-3** | **4+** |
| --- | --- | --- | --- | --- |
| White British/Irish | 22 679 | 89 | 7 | 5 |
| Caribbean | 399 | 64 | 18 | 18 |
| African | 436 | 79 | 12 | 10 |
| Indian | 1 012 | 85 | 10 | 5 |
| Pakistani | 877 | 76 | 15 | 9 |
| Bangladeshi | 453 | 66 | 24 | 10 |

Supplementary Table 4: Association of food insecurity with vegetable and fruit intake at wave 13

|  | **Adjusted for age and sex** | | **+ethnic group** | | **+ net income and IMD** | |
| --- | --- | --- | --- | --- | --- | --- |
|  | **OR** | **(95% CI)** | **OR** | **(95% CI)** | **OR** | **(95% CI)** |
| **Vegetables (n=24 161)** |  |  |  |  |  |  |
| 0 | 1 |  | 1 |  | 1 |  |
| 1-3 | 0.59 | (0.49, 0.70) | 0.60 | (0.50, 0.72) | 0.67 | (0.56, 0.81) |
| 4+ | 0.31 | (0.24, 0.40) | 0.31 | (0.24, 0.41) | 0.37 | (0.28, 0.49) |
|  |  |  |  |  |  |  |
| **Fruit (n=24 171)** |  |  |  |  |  |  |
| 0 | 1 |  | 1 |  | 1 |  |
| 1-3 | 0.63 | (0.52, 0.76) | 0.63 | (0.52, 0.77) | 0.69 | (0.57, 0.84) |
| 4+ | 0.29 | (0.22, 0.38) | 0.29 | (0.22, 0.38) | 0.34 | (0.25, 0.45) |

Supplementary Figure1 : Estimated ORs for each ethnic group (compared to baseline of wave 1 for white British/Irish) from longitudinal models for vegetable intake from waves 2, 5, 7, 11, and 13 of Understanding Society (estimated for 50 year old male)

1. adjusted for age, sex, b) adjusted for age, sex, income and IMD


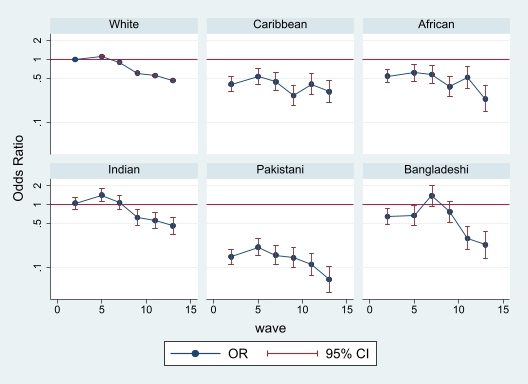

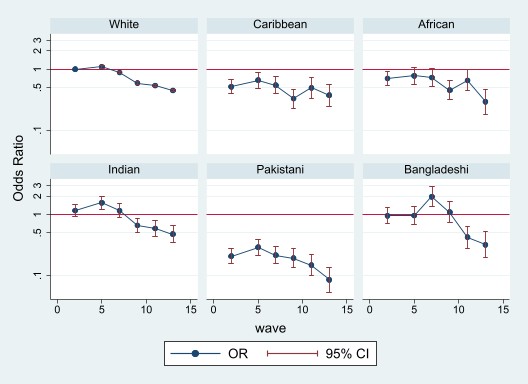


Supplementary Figure 2 : Estimated ORs for each ethnic group (compared to baseline of wave 1 for white British/Irish) from longitudinal models for fruit intake from waves 2, 5, 7, 11, and 13 of Understanding Society (estimated for 50 year old male)

1. adjusted for age, sex, b) adjusted for age, sex, income and IMD


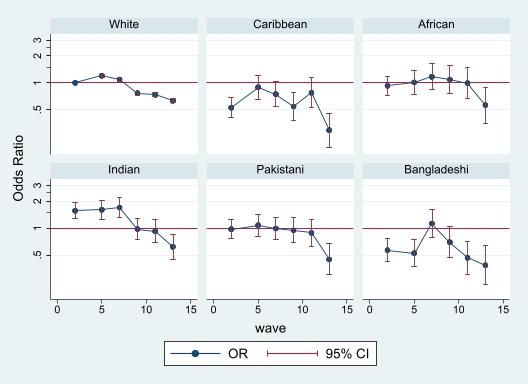

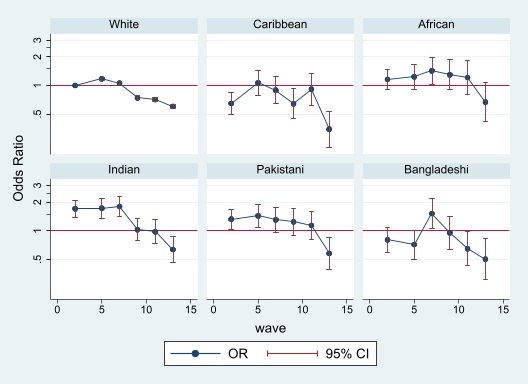


Supplementary Table 5. Multilevel logistic regression from wave 7 with binary outcome (including immigration and ethnic boost sample IEMB)

|  | **Vegetables (n=37, 644)** | | | | **Fruit (n=37, 641)** | | | |
| --- | --- | --- | --- | --- | --- | --- | --- | --- |
|  | **Adjusted for age and sex** | | **+net income and IMD** | | **Adjusted for age and sex** | | **+net income and IMD** | |
|  | **Reg Coeff** | **(95% CI)** | **OR** | **(95% CI)** | **OR** | **(95% CI)** | **OR** | **(95% CI)** |
| **Wave 7 (baseline)** |  |  |  |  |  |  |  |  |
| White British/Irish | 1 |  | 1 |  | 1 |  | 1 |  |
| African | 0.42 | (0.32, 0.55) | 0.56 | (0.42, 0.74) | 0.75 | (0.58, 0.99) | 0.96 | (0.73, 1.25) |
| Caribbean | 0.54 | (0.42, 0.71) | 0.76 | (0.59, 0.99) | 1.21 | (0.95, 1.56) | 1.61 | (1.25, 2.06) |
| Indian | 0.87 | (0.69, 1.08) | 1.00 | (0.80, 1.24) | 1.33 | (1.08, 1.64) | 1.51 | (1.23, 1.86) |
| Pakistani | 0.15 | (0.12, 0.20) | 0.22 | (0.16, 0.29) | 0.92 | (0.73, 1.15) | 1.26 | (1.00, 1.59) |
| Bangladeshi | 1.39 | (0.99, 1.94) | 2.13 | (1.52, 2.98) | 0.92 | (0.67, 1.27) | 1.33 | (0.97, 1.83) |
|  |  |  |  |  |  |  |  |  |
| **Linear change over time (per year)** |  |  |  |  |  |  |  |  |
| White British/Irish | 0.894 | (0.887, 0.902) | 0.891 | (0.884, 0.899) | 0.908 | (0.900, 0.916) | 0.904 | (0.896, 0.912) |
| African | 1.040 | (0.979, 1.105) | 1.038 | (0.977, 1.102) | 0.976 | (0.919, 1.104) | 0.975 | (0.918, 1.037) |
| Caribbean | 1.003 | (0.944, 1.066) | 1.000 | (0.942, 1.063) | 1.012 | (0.953, 1.075) | 1.011 | (0.952, 1.073) |
| Indian | 0.934* | (0.894, 0.975) | 0.929* | (0.890, 0.970) | 0.918* | (0.879, 0.958) | 0.916* | (0.877, 0.956) |
| Pakistani | 0.944^ | (0.891, 1.001) | 0.941* | (0.888, 0.998) | 0.962 | (0.915, 1.011) | 0.962 | (0.916, 1.010) |
| Bangladeshi | 0.840* | (0.785, 0.899) | 0.843* | (0.788, 0.902) | 0.947 | (0.883, 1.015) | 0.950 | (0.886, 1.018) |

*p<0.05 compared with white British/irish, ^0.05<p<0.1 compared with white British/Irish
